# Teaching clinical reasoning through hypothetico-deduction is (slightly) better than self-explanation in tutorial groups: An experimental study

**DOI:** 10.1007/s40037-018-0409-x

**Published:** 2018-02-26

**Authors:** Ahmed Al Rumayyan, Nasr Ahmed, Reem Al Subait, Ghassan Al Ghamdi, Moeber Mohammed Mahzari, Tarig Awad Mohamed, Jerome I. Rotgans, Mustafa Donmez, Silvia Mamede, Henk G. Schmidt

**Affiliations:** 1 College of Medicine, King Saud bin Abdulaziz University of Health Sciences, Ryiadh, Saudi Arabia; 2 Imperial College London, Lee Kong Chian School of Medicine, Nanyang Technological University, Singapore, Singapore; 3 Department of General Practice, Erasmus Medical Center, Rotterdam, The Netherlands; 4 Institute of Medical Education Research Rotterdam, Erasmus Medical Center, Rotterdam, The Netherlands; 5 Department of Psychology, Erasmus University Rotterdam, Rotterdam, The Netherlands; 6 Lee Kong Chian School of Medicine, Nanyang Technological University, Singapore, Singapore

**Keywords:** Clinical teaching, Clinical reasoning, Self-explanation, Hypothetico-deductive

## Abstract

**Background:**

Self-explanation while individually diagnosing clinical cases has proved to be an effective instructional approach for teaching clinical reasoning. The present study compared the effects on diagnostic performance of self-explanation in small groups with the more commonly used hypothetico-deductive approach.

**Methods:**

Second-year students from a six-year medical school in Saudi Arabia (39 males; 49 females) worked in small groups on seven clinical vignettes (four criterion cases representing cardiovascular diseases and three ‘fillers’, i.e. cases of other unrelated diagnoses). The students followed different approaches to work on each case depending on the experimental condition to which they had been randomly assigned. Under the self-explanation condition, students provided a diagnosis and a suitable pathophysiological explanation for the clinical findings whereas in the hypothetico-deduction condition students hypothesized about plausible diagnoses for signs and symptoms that were presented sequentially. One week later, all students diagnosed eight vignettes, four of which represented cardiovascular diseases. A mean diagnostic accuracy score (range: 0–1) was computed for the criterion cases. One-way ANOVA with experimental condition as between-subjects factor was performed on the mean diagnostic accuracy scores.

**Results:**

Students in the hypothetico-deduction condition outperformed those in the self-explanation condition (mean = 0.22, standard deviation = 0.14, mean = 0.17; standard deviation = 0.12; *F*(1, 88) = 4.90, *p* = 0.03, partial *η*^2^ = 0.06, respectively).

**Conclusions:**

Students in the hypothetico-deduction condition performed slightly better on a follow-up test involving similar cases, possibly because they were allowed to formulate more than one hypothesis per case during the learning phase.

## What this paper adds

Medical education places much value on the development of students’ diagnostic competence. Many schools now offer clinical reasoning courses early in the curriculum, but there is little empirical research on the approaches commonly used for the teaching of clinical reasoning. This experiment compared the effectiveness of two teaching approaches: self-explanation and hypothetico-deduction. The latter asks students to hypothesize about plausible diagnoses for clinical findings that are presented sequentially. Despite being very common, its effectiveness has rarely been investigated. The hypothetico-deduction approach worked slightly better than self-explanation to foster students’ diagnostic performance. Possible explanations for the findings are discussed.

## Introduction

The acquisition of competence in the skill of diagnostic reasoning is perhaps the most important task a medical student is confronted with, a task that is fraught with difficulties. Not only does the student have to learn to distinguish between 700+ different diseases, these diseases tend to present in quite idiosyncratic ways in patients. In addition, contextual influences, such as time pressure [1], patients’ disruptive behaviours [2] and a variety of cognitive biases such as availability bias [3], seem to add to the difficulty of arriving at the right diagnosis. The teaching of clinical reasoning is therefore an inherently challenging endeavour.

Teaching clinical reasoning has been traditionally left to the clinical rotations, intuitively the best place to learn these skills. However, this maxim is not true to the same extent as it was for a long time, as research findings and anecdotal evidence suggest [4]. Supervision and feedback are often suboptimal in clinical rotations, and students tend to be exposed to a patient population that does not replicate the range of health problems that they will encounter in professional life [5]. In response to these developments, medical schools have begun to establish clinical reasoning courses earlier in the curriculum, during which students become acquainted with the art and science of diagnostic reasoning by practising with clinical problems. Early examples concern employing simulated patients for this task. More recent additions involve the use of high-fidelity virtual patients presented online. Both are however expensive to develop and execute and have uncertain advantages over paper vignettes [6, 7]. Written clinical cases have therefore been extensively used, with a variety of instructional approaches being employed to teach clinical reasoning. Schmidt and Mamede have recently reviewed paper-based approaches that are currently used (or proposed) [4]. They distinguish between approaches on the basis of several dimensions, one of which is of interest for the present study: a distinction between cases unfolding in a sequential fashion (the ‘serial-cue’ approach) and ‘whole-case’ approaches. The basic difference between these two approaches is whether the case information is disclosed step-by-step or the entire case is available from the start. The former (‘serial-cue’) involves ‘hypothetico-deduction’, a way of reasoning resembling the diagnostic process of physicians [8]. Information about the patient only becomes sequentially available in the course of a student’s engagement with a case. Usually the patient’s chief complaint is presented and the students propose diagnostic hypotheses and deduce potential consequences, in terms of findings they would expect if the hypotheses were correct. Additional information is disclosed as the students progress through this process [9–11]. On the other hand, the ‘whole-case’ approach presents the case in full before students become involved with it. Schmidt and Mamede’s review [4] seems to suggest that whole-case approaches are generally more effective than serial-cue approaches. However, their evidence was based on a limited number of studies [12].

Among the whole-case approaches, a rather promising method in the teaching of clinical reasoning is *self-explanation* [13]. Chamberland and colleagues [14, 15] presented cases to advanced students and asked them, in addition to diagnosing these cases, to explain the signs and symptoms in terms of their underlying pathophysiology. A control group was simply asked to diagnose the same cases. The aim was to investigate whether self-explanation would foster students’ ability to distinguish between diseases that could explain a particular clinical presentation (for example, possible diagnoses for a patient presenting with chest pain and shortness of breath). The assumption underlying self-explanation was that by reactivating pathophysiological knowledge previously learned, the pathophysiological explanation would act as the underlying fabric more clearly tying together the signs and symptoms of these cases [16]. This would in turn lead to better diagnostic performance on similar cases presented at a later date. Chamberland found evidence showing just that, however only for cases that the students were not very familiar with.

The Chamberland studies presented cases to individual students to assess their impact. Such an approach, while theoretically important, is not amenable to introduction into an actual medical curriculum. In the Chamberland studies, for instance, each student had his or her own facilitator, who was tasked with encouraging the student to think aloud while dealing with the case. In actual programs, however, students would probably be practising in the presence of peers or in small groups. What would happen if *groups* of students were to work on cases? There is much evidence that having groups of students collaborate adds to the individual members’ learning and performance [17]. Such superior performance emerges because groups encourage individual students to elaborate on their prior knowledge (which facilitates further learning) and in addition to learn from each other.

The purpose of the present study, then, was to assess the effects of a self-explanation approach in small groups, relative to a hypothetico-deductive approach, on students’ performance in the diagnosis of the same clinical cases. Based on the previous studies by Chamberland and a study by Nendaz [12], our hypothesis was that the self-explanation group would do better than the hypothetico-deduction group on a test with similar cases. To test this hypothesis, groups of six students either processed seven cases through self-explanation or via hypothetico-deduction. One week later, they were presented with eight new cases (four of which were directly relevant to the cases processed during the previous learning phase) which they had to diagnose. The mean number of accurate diagnoses was taken as a measure of the quality of learning taking place in the learning phase.

## Method

### Design

The study consisted of two phases: a learning phase and a delayed diagnostic performance test administered 1 week later. In the learning phase, participants in small groups of approximately six discussed and diagnosed seven clinical cases under two different experimental conditions. Students were randomly assigned to the conditions of the experiment. Students in the *hypothetico-deduction* condition were presented with case information in a sequential fashion, had to provide tentative hypotheses, test these hypotheses as more information became available, and discuss their findings in small groups. The students in the *self-explanation* condition were presented with the whole case and were asked to explain the signs and symptoms in terms of their underlying pathophysiology in small groups, and provide a diagnosis as well.

The test required candidates to diagnose a set of eight new clinical cases, of which four criterion cases represented new exemplars of the clinical presentations encountered in the learning phase and four represented “fillers” (cases of different diseases used to decrease the chance that participants would easily recognize the new set of cases as representing the diseases seen in the learning phase).

#### Participants

All 188 second-year medical students at King Saud bin Abdulaziz University Medical College, in Riyadh, Saudi Arabia, a six-year medical school, were invited to participate in this study. We recruited Year 2 students because, at this point in their training, they have been exposed to theoretical knowledge about diseases but not yet seen any patients. Written consent was obtained from all students involved. They were promised that data would be analyzed anonymously.

Ethical approval for the study was given by King Abdullah International Medical Research Center (KAIMRC) Riyadh, Kingdom of Saudi Arabia. The study was carried out in accordance with the Declaration of Helsinki.

### Materials

Two sets of different clinical cases were used in the study, one for each phase (See Tab. 1 for the diagnoses involved). Each case consisted of a half-page description of a patient’s medical history, present complaints, findings of a physical examination and results of laboratory tests. See Tab. 2 for an example of such a case. The cases were based on real patients and had been used in previous studies [15]. Part of the cases consisted of cardiovascular diseases, another part of unrelated diseases (filler cases). The former were the criterion cases to be considered for the primary analysis (because the instructional approaches aim at increasing students’ ability to distinguish between diseases that are part of the differential diagnosis of a particular clinical presentation). The latter were included to reinforce the idea that both learning and test phase were clinical reasoning exercises and are therefore not relevant for the primary analysis.

**Table 1:** Diagnoses of the cases used in the different phases of the study

Learning phase	Test phase
–	Case 2.0 —Stomach cancer (Filler)
Case 1.1—Acute myocardial infarction with heart failure	Case 2.1—Chronic CAD, with decompensated heart failure by anaemia
Case 1.2—Community-acquired pneumonia (Filler)	Case 2.2—Acute pyelonephritis (Filler)
Case 1.3—Aortic stenosis with heart failure	Case 2.3—Chronic mitral insufficiency with secondary heart failure
Case 1.4—Nephrotic syndrome (Filler)	Case 2.4—Meningoencephalitis (Filler)
Case 1.5—Hypertensive cardiomyopathy	Case 2.5—Hypertensive cardiomyopathy
Case 1.6—Acute viral hepatitis (Filler)	Case 2.6—Acute appendicitis
Case 1.7—Alcoholic cardiomyopathy	Case 2.7—Viral myocarditis
–	Case 2.8—Rheumatoid arthritis (Filler)

**Table 2:** A case of acute myocardial infarction with heart failure

A 59-year-old businessman presents in the emergency department with severe dyspnoea. For the last 2 months, the patient has noted increasing shortness of breath: at first on climbing the stairs, and since last week at the least effort. The last two nights were particularly difficult, the patient experiencing shortness of breath even when lying down which forced him to sleep sitting up in a chair. He did not notice any cough or sputum. He used a salbutamol inhaler, which he uses as needed for asthma, without result. In the last 24 h he has also noted 4–5 episodes of tightness of the chest, of moderate intensity, lasting 5 to 10 min. No palpitations or syncope. He had a cold last week, which resolved spontaneously. Medical history: Hypertension for some 20 years, apparently well controlled with diltiazem 240 mg daily. Seasonal asthma, for which he periodically takes steroids, using a dosing inhaler, and salbutamol. The patient smokes ½ pack of cigarettes/day; he reports a healthy diet	
Physical examination:	BP 100/60, steady pulse 105/min; the patient is clammy; RR 28/min, dyspnoea at rest with saturation of 88% on arrival—ambient air—and 92% using nasal cannula at 2 l/min; oral temperature 36.5. Jugular veins not distended. Heart sounds are normal, with presence of a B3. Presence of a systolic murmur noted, 2/6 at the apex radiating towards the armpit. On pulmonary examination, crackles noted bilaterally in the lower thirds and wheezes noted on expiration. The abdomen is normal. The lower limbs are normal
Laboratory results:	Blood count, electrolytes, creatinine and glycaemia are normal. The ECG shows q waves (inferior) and inversion of the T wave from V2 to V6 with displacement of 2 mm in V3, V4, V5. Elevated troponins, 0.12. Chest X‑ray showed perihilar haze, septal lines and a slight right pleural effusion

### Procedure

*Learning phase.* The learning phase required the students to diagnose seven clinical cases. The cases were presented through PowerPoint slides in one of two randomized orders. Participants were randomly assigned to either the self-explanation condition or the hypothetico-deduction condition by using the list of students enrolled in the second year of the program. Subsequently, they were subdivided in groups of six, each with a facilitator who was a member of the academic staff. Prior to the study, the facilitators attended a 2-hour training session aimed at familiarising them with the study and ensuring uniformity of the procedure. The facilitator’s task was to take care that the procedure as described below was followed meticulously. He or she did not provide feedback or otherwise interfere with the learning. Each student was also presented with a response booklet with blank pages in which he or she was asked to make notes.

In the self-explanation condition, once the case was presented, the students were given the following instructions: 1) Please read the case quickly. 2) Write down here one or more diagnoses that come to mind. 3) Write down in bullet points which pathophysiological process may have caused the signs and symptoms in this case. 4) Now discuss your ideas about the pathophysiology of the case with your colleagues. 5) What is your final diagnosis? The first three steps were taken individually. In step 4, students had to explain to each other how the signs and symptoms in the case were produced by the underlying pathophysiology. In step 5, they were to agree on a most likely diagnosis. After having reached an agreement, the next case was presented on screen. Students did not receive feedback. The steps taken individually required written responses, whereas the other steps demanded only verbal reporting.

In the hypothetico-deduction condition, each case was presented in sequential fashion: history, physical examination, and laboratory test results would appear only after students followed the relevant parts of the instructions: History: 1) Write down here one or more diagnoses that come to mind while reading the history. 2) What further information would you need to test these diagnostic hypotheses? 3) Now discuss your ideas with you colleagues. Physical examination: 4) Write down here one or more diagnoses that come to mind while reading the physical examination information. 5) What further information would you need to test these diagnostic hypotheses? 6) Now discuss your ideas with you colleagues. Laboratory tests: 7) Write down here one or more diagnoses that come to mind while reading the laboratory data. 8) What is your final diagnosis? 9) Now discuss this conclusion with your colleagues. Steps 3, 6, and 9 required students to discuss ideas with their colleagues; the other steps were taken individually. As in the self-explanation condition, the steps taken individually required written responses, whereas the other steps demanded only verbal reporting. After completing a case, the next case was presented sequentially. Students were allowed to take as much time as they needed, but facilitators were instructed to spend no more than an hour on the seven cases. Time was maximized for each case in each condition. No significant differences in time emerged.

*Test phase.* One week after the training phase, the students received, under examination conditions, a booklet with eight cases, four of which described a cardiovascular condition (criterion cases) and five were filler cases. Students were requested to read each case and write down the most likely diagnosis. At the end of the test phase, students were debriefed with regard to the purpose of the experiment.

### Data analysis

The diagnoses provided by the participants for the criterion cases in the test phase were evaluated as correct, partially correct or incorrect, receiving scores of 1, 0.5, or 0 respectively. The diagnosis was considered correct whenever the core correct diagnosis of the case was provided (e.g. ‘myocarditis’ in a case of viral myocarditis). When the core diagnosis was not given, but one component of the diagnosis was mentioned, the diagnosis was considered partially correct (e.g. ‘mitral insufficiency’ in a case of chronic mitral insufficiency with secondary heart failure). When the participant’s response did not fall into one of these categories, the diagnosis was considered incorrect. Three experts in internal medicine (G.A.C., M.M.M., and M.D.) independently evaluated participants’ responses for each case. Responses had been previously transcribed from the booklets to excel sheets so that evaluators were not aware of the experimental condition under which the diagnoses had been provided. Their evaluations corresponded for 89% of the diagnoses; discrepancies were resolved by discussion.

For each participant, the scores obtained on the four cases of cardiovascular diseases were averaged. An ANOVA (significance level: 0.05) with experimental condition (self-explanation versus hypothetico-deduction condition) as between-subjects factor was conducted. This analysis tested the hypothesis that self-explanation while solving clinical cases would foster learning and would lead to better diagnostic performance on the test.

## Results

Fifty-nine (out of 188) students declined and 41 students either failed to complete all the phases or provided insufficient data to be included. Eventually, 49 female and 39 male students (mean age 22.1 years, standard deviation 1.98) participated in the study.

Tab. 3 contains the descriptive statistics of the experiment.

**Table 3:** Means and standard deviations of diagnostic accuracy scores under the conditions of the experiment (self-explanation versus hypothetico-deduction) for male and female participants

Experimental condition	Mean	Standard deviation	*N*
Hypothetico-deduction	0.22	0.14	45
Self-explanation	0.17	0.12	43
Total	0.20	0.13	88

A univariate analysis of variance was conducted with experimental condition as independent variable and diagnostic accuracy as the dependent variable. Students in the hypothetico-deduction condition performed better than those in the self-explanation condition, *F*(1, 86) = 4.20, *p* = 0.04. The effect size was small (Cohen’s *d* = 0.38) [18].

No differences in performance were observed on the filler cases, *F*(1, 86) = 0.91, *p* = 0.76, Cohen’s *d* = 0.05, suggesting that the groups were indeed similar and randomization was successful.

## Discussion

The purpose of the present experiment was to study the effectiveness of self-explanation of clinical cases in small tutorial groups relative to a hypothetico-deductive approach. Our hypothesis was that the self-explanation approach would yield higher gains because it enables students to activate previously acquired pathophysiological knowledge that would create coherence among the signs and symptoms to be explained and therefore facilitate subsequent diagnosis of similar cases [15, 19]. To study this hypothesis, we required students to work in small groups on seven cases to either provide a suitable pathophysiological explanation for each of them, in addition to providing a diagnosis, or to hypothesize about signs and symptoms presented sequentially. One week later, all students received the same eight cases and were required to provide a diagnosis.

Contrary to expectation, the students who were asked to engage in hypothetico-deduction, a task very similar to the task of the physician, performed significantly better than the self-explanation group. The effect was small, but it should be realized that it emerged even though the two approaches were employed in a single session and a small number of cases. In real educational programs, the approaches would be repeatedly employed throughout a series of sessions and cases, with a potentially higher effect. This is somewhat surprising because self-explanation, and other interventions that aim at elaboration or strengthening a person’s knowledge base, are usually successful in doing so. Since arriving at a correct diagnosis is a knowledge-based activity, self-explanation should be expected to be helpful. This finding also seems to contradict previous findings by Chamberland and colleagues [15]. They found self-explanation to be the superior approach when measuring performance on a set of new cases at a later point in time. However, their learning phase entailed the interaction between a single student, rather than a group of students, and a facilitator. In addition, their control condition was not asked to process the cases sequentially, but to provide a best diagnosis based on the engagement with a whole case.

A number of possible explanations for these divergent findings present themselves.

First, some facilitators reported that students in the self-explanation groups had difficulty coming up with explanations incorporating mechanisms or principles underlying the signs and symptoms in the cases. It seemed that they had already forgotten much of the basic science they learned previously or had difficulty applying pathophysiology to actual clinical cases. This may be a reason that the self-explanation condition did not reach its full potential: it simply insufficiently activated relevant knowledge to strengthen the students’ knowledge base.

Second, the hypothetico-deductive condition encouraged students to explicitly consider more than one hypothesis, while the self-explanation condition did not. Since the cases in the learning phase and the test phase were not identical, the chances are that those who were hypothesizing about possible diagnoses also considered one of more diagnoses that returned in the test phase, giving them a slight edge over the self-explanation group. On the other hand, in the studies by Chamberland and colleagues [15] and those of Mamede and colleagues [20, 21], the knowledge elicitation procedures excelled in particular with transfer cases, that is: cases that were in the same domain (for instance: cardiovascular disease) but had different diagnoses. To be fair, it has to be noted, however, that their studies did not include a comparison with a hypothetico-deduction condition.

A third factor possibly favouring the hypothetico-deduction condition is that our experimental setup forced us to provide both groups with the same patient information, even if students in the hypothetico-deduction condition *did not ask for that information*. In real life, as in most educational settings, hypothetico-deduction is driven by the informational needs as seen by the doctor or student engaged in diagnosing a case. The problem-solver receives only the information he or she has asked for. Nendaz has demonstrated that, when doctors and students diagnose clinical vignettes using the hypothetico-deductive approach, they perform less well than groups who receive the whole case [12]. Our hypothetico-deduction condition may have profited from receiving all the information, even the information it did not ask for.

A fourth issue limiting our study is the surprisingly low performance of all our groups. With mean scores around 0.20 on a scale between 0 and 1, our participants’ achievements were well under the achievements of students in similar studies [20, 21]. Again, this may indicate that our participants simply did not yet have sufficient knowledge to deal with the cases, and therefore those who produced, perhaps haphazardly, more different hypotheses during the learning phase, had a slight edge over those who did not. More research is clearly necessary here.

It should be highlighted that many of these limitations can be seen as a side effect of our attempt at increasing ecological validity. We opted for comparing the two approaches under conditions that would closely simulate those encountered in an actual medical program: students worked in small groups with different facilitators. In doing so, we may have gained in validity, but strict control over the discussion in the groups was not possible. This comes as the unavoidable trade-off between ecological validity and experimental control.

In conclusion, the much-used hypothetico-deductive method for teaching clinical reasoning did relatively well in our study. Tentative explanations have been raised but further research is required to explore which approach works better and under which conditions. New methods, such as self-explanation, need further scrutiny.
